# MoGraphDRP: Multi-omics and graph fusion with bilinear attention for predicting drug sensitivity

**DOI:** 10.1371/journal.pone.0341458

**Published:** 2026-03-06

**Authors:** Zahra Ahmadi, Jamshid Pirgazi, Ali Ghanbari Sorkhi

**Affiliations:** Faculty of Electrical and computer Engineering, Department of Computer Engineering, University of Science and Technology of Mazandaran, Behshahr, Iran; The Catholic University of Korea, KOREA, REPUBLIC OF

## Abstract

Accurate prediction of drug response in cancer cells is a fundamental step toward achieving precision medicine and designing personalized therapies. In this study, a multi-branch deep learning framework is proposed that integrates multi-omics cellular data including gene expression, mutation, methylation, and biological pathways with structural features of drugs (molecular graphs and various chemical fingerprints) to enable drug response prediction. The graph structure of the drug is modeled using a three-layer Graph Convolutional Network (GCN), and chemical fingerprints are compressed using MLP networks. These multiple representations of drugs are integrated and then combined with cellular features in a Multi-head Bilinear Attention module to model the complex interactions between cells and drugs. In the final stage, an ensemble model based on XGBoost is used to refine the outputs. The MoGraphDRP model demonstrates significantly higher accuracy in drug response prediction compared to existing state-of-the-art methods. Experimental results show that the MoGraphDRP model outperforms advanced methods such as BANDRP, DeepCDR, and DeepTTA, achieving PCC = 0.9689, RMSE = 0.6622, and R² = 0.9388. This model not only accurately reconstructs missing IC50 values but also effectively distinguishes between sensitive and resistant drugs in unknown combinations. The MoGraphDRP framework can serve as a powerful, interpretable, and reliable tool for analyzing drug response and designing preclinical treatments.

## 1. Introduction

Cancer, as one of the most challenging and complex diseases of the modern era, causes the death of millions of people worldwide each year and imposes a significant economic and social burden on healthcare systems. This disease is not the result of a single factor, but rather the outcome of complex interactions among genetic, epigenetic, and environmental elements, which lead to severe heterogeneity among patients, even within the same cancer type [1,2]. Such heterogeneities, which manifest at the levels of gene expression, genetic mutations, and the tumor microenvironment, cause patients to respond differently and unpredictably to standard treatments such as chemotherapy, immunotherapy, or targeted therapies [2,3].

This reality presents a serious challenge to predicting treatment efficacy and has drawn special attention to treatment strategies based on “precision medicine.” Within this framework, determining the individual response of each patient to a specific drug aimed at optimizing treatment and reducing side effects has become a scientific and clinical priority [1,4]. However, traditional methods for drug discovery and evaluation, including in vivo (animal-based) and in vitro (cell line-based) experiments, are highly costly, time-consuming, and unscalable. Moreover, they are incapable of modeling the biological complexities of cancer cells in real environments [5]. Moreover, many anticancer drugs face the challenge of drug resistance a phenomenon often caused by the activation of alternative signaling pathways in tumor cells or by selective pressures induced by treatment [4]. In such cases, relying solely on gene mutation status or drug targets is insufficient for predicting drug efficacy and may lead to treatment failure or reduced patient survival rates [3].

Therefore, there is an urgent need for novel approaches to predict cancer cell responses to various drugs approaches capable of modeling the complexity of biological data and accurately predicting cellular responses to different drug compounds. This challenge has paved the way for the development of modern computational models in the field of Drug Response Prediction (DRP) [6,7]. With the advancement of biotechnologies, particularly the emergence of high-throughput genomics, it has become possible to collect and analyze multilayered data from cancer cells. These advancements have led to the creation of large public databases such as the Cancer Cell Line Encyclopedia (CCLE) [6] and Genomics of Drug Sensitivity in Cancer (GDSC) [7], which contain comprehensive information on genetic and epigenetic profiles, as well as drug responses of thousands of cancer cell lines. Additionally, the PubChem database, by cataloging the chemical structures of millions of drug compounds [8], provides a valuable resource for modeling the molecular structures of drugs. The availability of such data has laid a strong foundation for developing advanced computational models for drug response prediction. Over the past decade, numerous researchers have leveraged machine learning to design models aimed at predicting cancer cell sensitivity to drugs. Methods such as matrix factorization [9], Bayesian factorization [10], and network-based models relying on drug–cell similarity [11] represent some of the early efforts in this field. With the advancement of deep learning, more effective models have emerged that are capable of extracting hidden and complex features from biological data. For example, models such as CDRscan [12], which integrates drug fingerprints and gene mutations, Precily [13], which utilizes gene expression data, and RefDNN [14], which adopts deep neural network architectures, have made significant strides in drug response prediction. However, many of these models remain at lower levels of abstraction and do not exploit more natural representations of molecular drug structures such as chemical graphs. Furthermore, the use of genomic data in isolation, without accounting for functional relationships between genes, i.e., pathways can result in the loss of essential biological information [15–17].

In recent years, the rapid growth of deep learning and the emergence of specialized models for structural and biological data have fundamentally transformed the design of drug response prediction models. Unlike traditional models that represent drug data as strings (e.g., SMILES) or hand-crafted feature vectors, recent studies have utilized the graph structures of molecules to achieve a more natural representation of drugs [15,18]. Graph Neural Networks (GNNs) such as Graph Convolutional Networks (GCN), Graph Attention Networks (GAT), and Graph Isomorphism Networks (GIN) have been widely used for learning from molecular graphs, successfully extracting chemical structures and atomic interactions of drugs with higher precision [19–21]. In models like DeepCDR [22], drug graph information has been integrated with multi-omics data from cancer cells, yielding notable results. On the other hand, the use of attention mechanisms in models such as DeepTTA [23] and GraTransDRP [24] has shown that Transformer-based models can better capture long-range dependencies and complex interactions between cell and drug features.

Moreover, the focus on multimodal models those that combine drug fingerprints, molecular graph data, and genetic/epigenetic features of cells into a unified framework has significantly improved predictive accuracy. For instance, the GPDRP model [25] integrates molecular drug graphs with pathway activity scores, and the BANDRP model [26] employs a bilinear attention network to learn interactive representations between drug fingerprints and cellular omics data, both taking valuable steps in this direction. Despite these advances, challenges remain. Many models either use structural drug information incompletely or struggle with learning simultaneous interactions among data sources (such as fingerprints, graphs, and omics). Furthermore, in most models, the biological interpretation of gene function through pathways remains superficial, limiting the interpretability of the models [17,27]. Numerous studies have been conducted with the goal of improving drug sensitivity prediction in cancer cell lines using deep learning. In one study, Liu et al [28] employed deep convolutional neural networks to extract high-level features from omics data. The results demonstrated that CNN-based architectures are capable of identifying nonlinear patterns related to drug response. In another study, Chawla et al [29] presented a gene expression-based framework that inferred drug sensitivity using extensive transcriptomic data. Their findings indicate that gene expression information alone can have strong predictive power, especially when integrated with biological knowledge.

Additionally, Wang et al [30] introduced the GADRP model, which combines graph convolutional networks (GCNs) and autoencoders to simultaneously model drug chemical structures and multi-omics data of cancer cells. This approach, leveraging both graph-based and latent representations, outperformed traditional methods. Despite all the recent advances in drug response modeling, there remains a need for more precise, interactive, and interpretable frameworks. A significant portion of the current challenges can be summarized in three main areas:

First, many models rely solely on a single type of drug representation, such as SMILES strings or classical fingerprints, while ignoring the complete molecular structure of drugs, which can result in the loss of critical chemical information. In contrast, representing drugs as molecular graphs enables the modeling of atomic relationships and precise structural features [15,21].

Second, multi-omics information from cancer cells including gene expression, mutations, methylation, and biological pathway activity is often used incompletely or in isolation, without effective synchronization or interactive integration with drug features. However, accurate integration of these data can yield a rich and meaningful representation of the cell’s biological state [22,25,26].

Third, in many frameworks, the relationships between drug and cell features are modeled in a superficial or linear manner. Yet, biological interactions in the context of cancer are highly complex, multi-layered, and non-trivial. To model these relationships, attention-based architectures especially more advanced forms such as bilinear attention or graph-based attention mechanisms (Graph Transformers can offer significant advantages [23,24,26].

In this context, designing a model that can simultaneously leverage drug fingerprints and graph structures, enrich omics data through pathway understanding, and learn drug–cell relationships in an interactive and interpretable way could be a significant step forward in improving prediction accuracy, enhancing biological interpretability, and increasing the clinical applicability of DRP models. In response to these challenges, a hybrid, multi-branch framework is proposed that simultaneously utilizes drug fingerprints and graphs, multi-omics cellular data, and a dual-attention mechanism. The model architecture consists of specialized encoders, a Bilinear Attention module for learning feature-level interactions, and a refinement stage based on XGBoost. This study aims to design an integrated architecture capable of learning conditional interactions between drugs and cells and using fusion vectors for final prediction refinement. The remainder of the paper presents the architectural details, experimental results, comparative analyses, and model applications.

## 2. Proposed method of MoGraphDRP

**Fig 1:**
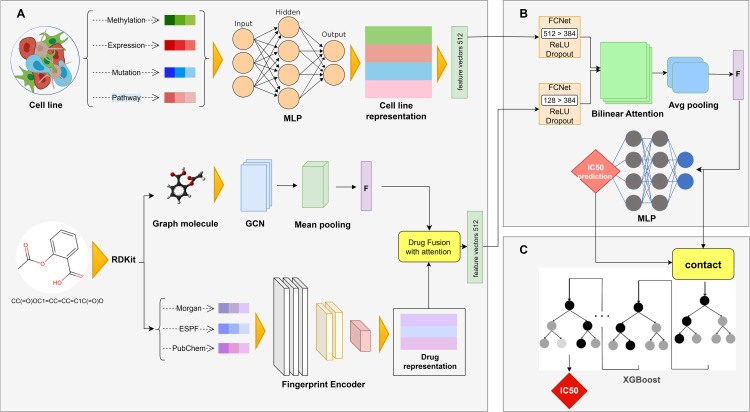
Overall architecture of the MoGraphDRP model for drug response prediction through the integration of cellular multi-omics data and composite drug structure representation. **(A)** Cellular omics data are processed separately through fully connected layers and ultimately fused into a compressed vector within the cell feature encoder module. Simultaneously, drug representations are extracted through two pathways: the first employs a molecular graph derived from SMILES and a GCN-based graph network; the second compresses chemical fingerprints (Morgan, ESPF, and PubChem) within the drug feature encoder module. The outputs of both pathways are merged using an attention-based integration module to form the final drug representation. **(B)** The final vectors of the drug and the cell are fed into the Bilinear Attention module. This module, leveraging multiple attention heads, models feature-level interactions between the two vectors and produces an enriched vector denoted as *f*, which represents the nonlinear drug–cell interaction. This vector is passed to a prediction network composed of multilayer perceptrons (MLPs) to estimate the IC50 value. **(C)** In the final stage, the initial predicted output *y*, along with the interaction vector *f*, is passed into an XGBoost-based boosting model that acts as the final refinement module to enhance prediction accuracy.

In this study, a multimodal deep learning framework is designed to accurately predict drug response in cancer cells. The overall architecture is illustrated in Fig 1. Inspired by the biological complexity of drug–cell interactions, this architecture aims to provide a comprehensive and learnable representation of each drug–cell pair by integrating multilayered omics data of the cell and structural features of the drug. In the cell line input module, four types of data gene expression, genetic mutations, DNA methylation, and biological pathway activity scores are used to capture various dimensions of cellular function. These independent profiles enable the model to simultaneously consider both low-level molecular and high-level functional aspects of the cell’s biological state. To compute the pathway activity scores, Gene Set Variation Analysis (GSVA) [31] is employed. This method estimates biological pathway activity based on gene expression without requiring labeled data or assuming a specific statistical distribution. In the drug representation module, a multi-branch architecture is designed to comprehensively extract chemical features through two complementary paths. In the first path, SMILES strings are converted into molecular graphs using the RDKit tool [32]. Then, topological features of the molecule are extracted using Graph Convolutional Networks (GCNs). This path allows the model to understand the global structure of the drug and model atomic relationships graphically. The second path focuses on low-level vector representations of drug structure and utilizes three complementary types of chemical fingerprints: Morgan [33], Explainable Substructure Partition Fingerprint (ESPF) [34], and PubChem. Each fingerprint provides distinct structural information: Morgan identifies local patterns and chemical rings, ESPF extracts electronic and pharmacophoric features, and PubChem models predefined substructures.

The combination of these three complementary representations aims to enhance the model’s ability to deeply understand the drug’s chemical structure. To compress and extract higher-level features from these representations, a multi-layer autoencoder is employed as the encoder. This hybrid approach produces a multidimensional and precise representation of the drug, which can significantly improve the performance of drug–target interaction prediction models. In the next step, the feature vector extracted from the molecular graph and the compressed fingerprint-based vector are fused using an attention-based integration module to produce a unified and enriched drug representation. This final drug representation is then combined with the cell vector, obtained from the multi-omics encoder, within a Bilinear Attention module. The structure of this module is based on a Bilinear Attention Network (BAN) combined with Multi-head Bilinear Pooling, enabling precise modeling of feature-level and complex nonlinear interactions between cellular and drug information. The output of this stage consists of two components: first, the initial predicted drug response value (IC50), and second, the compressed interaction vector (Fusion Vector) representing the degree and nature of synergy between drug and cell information.

In the final stage, to enhance prediction accuracy and robustness, both outputs are fed into an XGBoost-based boosting model. This stage, designed as an ensemble module, aims to correct residual errors and improve the overall performance of the model. This two-stage design (combining deep learning and boosting models) integrates the advantages of both approaches, allowing simultaneous utilization of the expressive power of neural networks in learning nonlinear relationships and the systematic error reduction capabilities of ensemble models. The MoGraphDRP model architecture is described in a modular fashion in the following sections.

### 2.1. Feature extraction from cell line

In complex biological systems such as cancer, the behavior of cell lines is influenced by multiple layers of biological information, each of which alone is insufficient to fully explain the drug response of a cell. Therefore, this model utilizes a multi-branch encoder designed to extract a compact and rich representation of the biological state of each cell line. The objective of this encoder is to generate a vector that captures both the molecular and functional aspects of the cell, which can then be used in the subsequent stages of the model to learn drug–cell interactions.

To comprehensively represent the biological status of cancer cells, four types of omics data were used: gene expression, genetic mutations, DNA methylation, and biological pathway scores. Each of these branches captures a specific dimension of cellular biology: the gene expression branch is intended to identify regulatory patterns of genes associated with drug sensitivity, while the mutation branch emphasizes the presence or absence of mutations in key genes. The methylation branch extracts epigenetic information related to gene regulation, and the pathway branch provides a high-level, functional view of intracellular signaling system activity. This diversity of profiles allows the model to simultaneously understand and analyze both low-level molecular data and high-level biological functions. To this end, for each cell $c_{i}$, four types of omics data were collected from reputable sources: gene expression ${\;c}_{i}^{exp}\in R^{\text{7}\text{1}\text{4}}$, genetic mutations $c_{i}^{mut}\in R^{\text{7}\text{1}\text{5}}$, DNA methylation $c_{i}^{meth}\in R^{\text{6}\text{0}\text{3}}$, and biological pathway activity scores $c_{i}^{path}\in R^{\text{1}\text{2}\text{8}\text{3}}$. Pathway scores were calculated using the GSVA method [31] based on the C2 collection of pathways from the MSigDB database [35], utilizing gene expression data. After normalization and alignment with cancer-related genes based on the COSMIC database, these profiles were fed into the neural encoder network.

For each type of data, an independent learning branch is designed to specifically model the unique statistical and semantic structure of that data type. Each branch consists of two fully connected layers, with batch normalization, ReLU activation, and dropout applied between them. This two-layer architecture enables the learning of nonlinear transformations and gradual dimensionality reduction, while also preventing overfitting. In the first stage, the raw vector of each biological data type is projected into an initial compact latent space:


$$h_{i}^{\left(v\right)}\;=\;ReLU\;({BN}^{\left(v\right)}\;(W_{\text{1}}^{\left(v\right)}a_{i}^{\left(v\right)}+h_{\text{1}}^{\left(v\right)}))$$ (1)

Subsequently, this representation is mapped into a shared latent space with a fixed dimension, where the vector length is set to *l = 128*. This value was selected based on empirical testing with different sizes (64, 128, 256) and provides a balanced trade-off between compression and the preservation of biological information:


$$t_{i}^{\left(v\right)}\;=\;ReLU\;(W_{\text{2}}^{\left(v\right)}h_{i}^{\left(v\right)}+b_{\text{2}}^{\left(v\right)})$$ (2)

In these equations, *v* ∈ *{exp, mut, meth, path}* denotes the type of biological profile, and $W_{\text{1}}^{\left(v\right)}$ and $W_{\text{2}}^{\left(v\right)}$ are the weight matrices of the first and second layers, respectively, whose dimensions are determined according to the input size and the latent space. The four compressed vectors obtained from each branch $t_{i}^{exp},\;t_{i}^{mut},\;t_{i}^{meth},\;t_{i}^{path}\in \;R^{\text{1}\text{2}\text{8}}$ are then combined in the final stage through concatenation along the feature dimension:


$$h_{i}^{cell}\;=t_{i}^{exp}⊕\;t_{i}^{mut}⊕\;t_{i}^{meth}⊕\;t_{i}^{path}\in \;R^{l}$$ (3)

Here, the symbol ⊕ denotes vector concatenation. The result is a unified 512-dimensional compressed vector representing the biological state of the cell. Instead of using attention-based or weighted fusion, direct concatenation was employed, since the number of biological profiles in this architecture is limited and independent. Initial experiments showed that concatenation provided more stable performance compared to methods that learn fusion weights. This combination is used in subsequent stages of the model particularly in integration with drug features within the Bilinear Attention module. This multi-purpose representation significantly enhances the model’s ability to learn complex drug–cell interactions.

### 2.2. Feature extraction from drug

Accurate prediction of drug response requires a deep understanding of the chemical structure of drugs. Since drug structures consist of multiple dimensions including topological information, pharmacophoric features, and recurring substructures relying on a single type of representation may lead to the loss of critical information. Therefore, in the MoGraphDRP model, the drug encoder is designed as a dual-branch architecture, comprising a chemical fingerprint branch and a molecular graph branch. Both branches originate from a common input and extract complementary representations of the drug’s structure.

For each drug, the SMILES string is provided as input to the RDKit tool [32]. This tool interprets the chemical structure and generates two independent representations: first, a molecular graph $G_{J}\;=(V_{J}\;,E_{j})$, where each node represents an atom and each edge denotes a chemical bond between atoms. This graph allows for precise modeling of the drug’s topological structure. Second, molecular fingerprint vectors are generated by compressing structural features of the drug into fixed-dimensional, neural-network-compatible vectors. These two types of representations are employed complementarily in the model architecture to capture various aspects of the drug’s chemical structure.

#### 2.2.1. Feature extraction based on fingerprints.

Molecular fingerprints are compact, standardized numerical representations of drug structures that can be efficiently utilized in neural networks. In this model, three types of complementary fingerprints were used to capture diverse structural features. The Morgan fingerprint, with 2048 dimensions, captures atom neighborhood patterns with a fixed radius and is suitable for representing local functional groups. The PubChem fingerprint, with 881 dimensions, is designed based on the presence or absence of predefined chemical substructures. Finally, the ESPF fingerprint, with 2586 dimensions, encodes bio-pharmacophoric features directly from SMILES strings using a structured encoding scheme. All fingerprints were directly extracted from the drug SMILES strings using the RDKit toolkit. Although fingerprints do not provide a holistic structural view, their compactness and computational efficiency make them highly effective for fast and interpretable learning. For each fingerprint type, a dedicated multi-layer encoder was designed to project the numerical data into a more compact latent space. These encoders consist of multiple fully connected layers with ReLU activation and dropout, dynamically configured based on the input dimension. The purpose of this process is to remove noise, extract high-level representations, and align the data with a common learning space. The output of each encoder is a 128-dimensional compressed feature vector.


$$h_{i}^{\left(k\right)}\;={ReLU\;(W}_{\text{2}}^{\left(k\right)}.\;Relu(\;W_{\text{1}}^{\left(k\right)}.\;f_{j}^{\left(k\right)}+\;b_{\text{1}}^{\left(k\right)})+\;b_{\text{2}}^{\left(k\right)})$$ (4)

Where k∈{Morgan,PubChem,ESPF}*,* and the weight parameters $W_{\text{1}}^{\left(k\right)}$ and $W_{\text{2}}^{\left(k\right)}$ are learnable. The output of each pathway is compressed to a dimension of *l*. Then, the three resulting compressed vectors are concatenated with equal weights.


$$h_{i}^{FP}\;=h_{j}^{Morgan}⊕\;h_{j}^{PubChem}⊕\;h_{j}^{ESPF}\in \;R^{\text{3}l}$$ (5)

#### 2.2.2. Feature extraction based on graph network.

**Fig 2:**
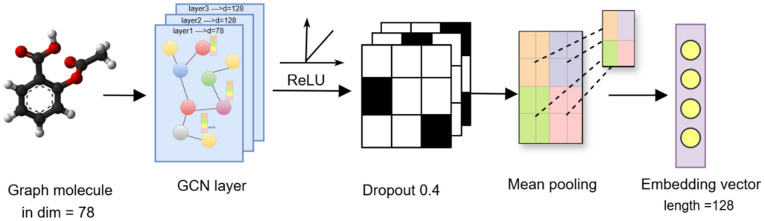
Architecture of the drug graph module based on GCN. First, the molecular graph with node feature vectors of dimension 78 is input into a three-layer graph neural network, where each layer employs message averaging, a ReLU activation function, and dropout with a rate of 0.4. Finally, using a mean pooling function, a fixed-length vector of dimension 128 is generated as the representation of the entire graph.


Each drug can be modeled as a molecular graph in which atoms are defined as nodes and chemical bonds as edges. This graph is extracted from the SMILES string using RDKit and preserves the topological structure of the drug, whereas fingerprints merely enumerate existing substructures. For this purpose, each drug is represented as an undirected graph $G_{j}=(E_{j}\;,V_{j})$, where $V_{j}$ is the set of nodes (atoms) and $E_{j}$ is the set of edges (chemical bonds). The features of each node are initialized using a vector that includes the chemical element type, number of bonds, aromaticity, number of hydrogen bonds, and other descriptive properties. To learn the structural representation of these graphs and extract features, a Graph Convolutional Network (GCN) is employed. In this architecture, a GCN is used to capture local features, as illustrated in Fig 2. This network extracts hierarchical local features of the molecule by passing messages between neighboring nodes and applying ReLU and dropout with a rate of 0.4 at each layer. After three rounds of message passing, a mean pooling function is applied across the nodes to generate a fixed-length vector representing the entire graph. The node feature update in each GCN layer is performed as follows:

$$h_{v}^{\left(t+\text{1}\right)}\;=ReLU(W^{\left(t\right)}.\;MEAN(\left\{h_{u}^{\left(t\right)}|u\in N(v)\right\}\cup \{h_{u}^{\left(t\right)}\}))$$ (6)

After passing through the message-passing layers, the final graph vector is computed using a mean pooling function over all nodes:


$$h_{i}^{GCN}\;=MEAN\;(\left\{h_{u}^{\left(t\right)}|\;u\in \;V_{j}\right\})$$ (7)

Where *T* is the total number of graph layers, and ${\;h}_{i}^{GIN}\in \;R^{l}$ represents the final vector representation of the drug. The use of GCN allows the model to learn the global structure of the drug and long-range interactions between atoms an aspect that fingerprints are unable to capture.

#### 2.2.3. Fusion of drug features.

Since the chemical structure of a drug can be described from various dimensions, relying solely on a single representation may overlook critical information. In this architecture, both fingerprints and graphs are used simultaneously. Molecular fingerprints (such as Morgan, PubChem, and ESPF) effectively model aspects of local structure, pharmacological features, and the presence of specific substructures, making them successful in quickly identifying substructures and pharmacophoric features. However, they lack awareness of the overall molecular structure. In contrast, molecular graphs focus on the topological interactions between atoms and the global structure of the molecule, but are computationally intensive and more difficult to interpret directly. To enable the model to obtain a more comprehensive view of the drug molecule, it is essential to integrate these two complementary representations. In the MoGraphDRP architecture, this integration is performed via simple vector concatenation. First, the compressed graph representation of the drug ${\;h}_{i}^{GCN}\in \;R^{l}$ and the fingerprint representation ${\;h}_{i}^{FP}\in \;R^{l}$ are generated. Then, these two vectors are concatenated along the feature dimension.


$$h_{i}^{Drug}\;=h_{j}^{GCN}⊕\;h_{j}^{FP}\in \;R^{\text{3}l}$$ (8)

Here, ⊕ denotes vector concatenation, and *l* is the dimensionality of the compressed graph representation space. The choice of concatenation is motivated by the fact that the number of branches is limited and each provides an independent representation. Based on various ablation studies, learning attention weights or gating at this level adds high complexity without providing significant improvement, and in empirical experiments, simple concatenation has shown more stable and generalizable performance. This fusion method enables the model to simultaneously leverage structural, local, and bio-pharmacological features of the drug. Since none of these representations alone captures all biological aspects of the molecule, their integration can enhance the model’s generalization capability to new and unseen drugs. This dual-branch model, by combining fast, interpretable, and structure-aware representations, offers a comprehensive depiction of the drug molecule while maintaining generalizability to novel data. The final vector $h_{i}^{Drug}$ serves as the complete drug representation and is used in the next stage for interaction with cellular features.

### 2.3. Bilinear attention module and drug–cell interaction modeling

One of the fundamental challenges in drug response modeling is understanding the complex, nonlinear, and multi-level interactions between the biological features of cancer cells and the chemical structures of drugs. In many existing architectures, the integration of these two types of information is performed using methods such as concatenation or dot-product attention. However, these approaches typically assume that the relationships between features are direct and linear, and thus are incapable of modeling indirect, combinatorial, and conditional interactions between the two independent spaces (cell and drug). To overcome this limitation, the MoGraphDRP model employs a bilateral attention module called Bilinear Attention, designed to learn independent and conditional feature-level interactions between two vectors. This module enables the model to allow each cellular feature to interact with a specific combination of drug features, and vice versa. Unlike conventional attention mechanisms, which typically operate based on overall similarity or inner product, bilinear attention utilizes a bilinear form, which offers greater capacity for modeling specific combinations of cross-domain features. In this module, the compressed vectors of the drug ${\;h}_{i}^{Drug}\in \;R^{\text{1}\text{2}\text{8}}\;$ and the cell ${\;h}_{i}^{Cell}\in \;R^{\text{5}\text{1}\text{2}}\;$ are each projected into a shared space $R^{\text{2}\text{5}\text{6}}$ via independent linear layers.


$${\tilde{h}}_{i}\;=\;W_{c}h_{i}^{Cell}+b_{c\;}\;,\;{\tilde{h}}_{j}\;=\;W_{c}h_{j}^{Drug}+b_{d}\;$$ (9)

$${\tilde{h}}_{i}\;,\;{\tilde{h}}_{j\;}\in \;R^{d}$$

Projection into a shared space is a prerequisite for interaction assessment, as the two input vectors originate from different domains and must reside in a semantically aligned and unified space to enable direct interaction. In the next step, the interaction between these two vectors is modeled using a learnable bilinear weight matrix $U\in R^{\text{2}\text{5}\text{6}*\text{2}\text{5}\text{6}}$ to compute the bilinear attention mapping:


$$I\;=\;{\tilde{h}}_{i}^{T}.U.{\tilde{h}}_{j}\;\in \;R\;$$ (10)

In this equation, the scalar value *I* represents the interaction strength between all features of the drug and the cell, taking into account nonlinear combination weights. To enhance discriminative power and capture various possible interactions, this structure is extended into a Multi-Head Bilinear Attention:


$$I_{k}\;=\;{\tilde{h}}_{i}^{T}.U_{k}.{\tilde{h}}_{j}\;,\;for\;k=\;\text{1},...,K\;$$ (11)

Where *K* is the number of attention heads, and each $U_{k}$ is an independent weight matrix for learning a specific type of interaction. This mechanism allows the model to simultaneously capture multiple distinct patterns of drug–cell associations. To extract an interaction vector from the output of the heads, the $I_{k}$ values are mapped to a compressed vector using a Fully Connected layer:


$$f_{ij}\;=\;ReLU(W_{f}\;.\;\left[\;I_{\text{1}}\|\;I_{\text{2}}\|\;...\|I_{K}\right]+b_{f})\in \;R^{\text{1}\text{2}\text{8}}\;$$ (12)

Where $f_{ij}$ is a feature-level drug–cell interaction vector that encapsulates compressed information from all attention heads. This vector serves as a bilinear fusion representation and is used not only for IC50 prediction but also in the subsequent stages of the model (ensemble module). To estimate the IC50 value, the fusion vector $f_{ij}$ produced by the Bilinear Attention module representing the combined features of drug and cell is fed into a multilayer perceptron (MLP). This network consists of two Fully Connected layers in the sequence: FC (128 → 64) → ReLU → Dropout → FC (64 → 1). It’s simple and lightweight structure is designed to prevent overfitting during the regression phase and enables the learning of a nonlinear mapping from the fusion vector space to drug response values:


$${\hat{y}}_{ij}\;=\;MLP(f_{ij})\;$$ (13)

**Fig 3:**
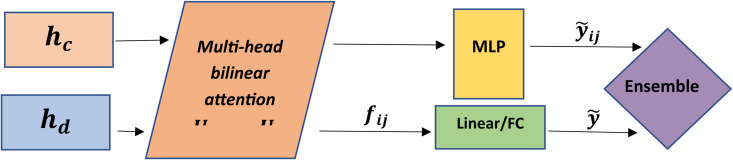
Structure of the Bilinear Attention module in the MoGraphDRP architecture. First, the compressed vectors of the cell and the drug are mapped into a shared space through linear layers. Then, feature-level interactions between them are learned via multi-head bilinear attention using the $U_{K}$ matrices. The output of this module consists of two parts: (1) the predicted IC50 value for the drug–cell pair, and (2) the compressed interaction vector $f_{ij}$, which contains the features extracted by the model. Both outputs are used as inputs for the final stage of the model the ensemble network to enhance prediction accuracy.

Where ${\hat{y}}_{ij}$ is the predicted drug response value (IC50) for the given drug–cell pair. In addition to directly predicting the IC50 value, the fusion vector $f_{ij}$ is also independently stored for the reinforcement learning stage and the ensemble model as a feature vector. Thus, the MLP acts as a preliminary predictor, optimized through end-to-end training to provide a raw but effective estimate of drug response, while the fusion output serves as a reinforcement vector for secondary refinement of results. This dual-path design improves accuracy and reduces sensitivity to noise in the final prediction stage. As shown in Fig 3, the Bilinear Attention module is designed to first map both vector spaces into a shared space, then compute multiple bilinear interactions, and finally compress them into a representation usable in the final prediction.

### 2.4 Ensemble regression head

Despite the high capability of the Bilinear Attention module in modeling drug–cell interactions and producing an initial estimation of drug response, the final prediction accuracy may still be affected by factors such as the complexity of biological relationships, data noise, or the inherent limitations of neural models. Especially in deep architectures with a large number of parameters, the model may fail to correctly adjust residual errors or detect subtle patterns. Therefore, in the final step of the MoGraphDRP architecture, a refinement module based on ensemble learning is designed to enhance the accuracy and robustness of IC50 prediction. At this stage, the XGBoost model is employed as a refinement layer. The choice of XGBoost is motivated by its high capability to model residual errors with complex nonlinear structures and its resilience against overfitting. Unlike models such as Random Forest or AdaBoost, this algorithm provides higher accuracy and better stability in noisy contexts such as biological data by leveraging regularization, second-order gradients, and fine control over model complexity. In contrast to typical ensemble models that replace the main network, XGBoost here plays a complementary role being applied to the outputs of the previous stage to reduce remaining errors. The XGBoost algorithm, through an ensemble of decision trees trained incrementally by following the error gradient, is highly effective at identifying residual patterns not captured by the main model. Each tree in this algorithm is of the CART (Classification and Regression Tree) type, where node splits are made to maximize the reduction in the loss function. These trees are capable of modeling nonlinear relationships and conditional interactions among features. The input to this module is a combination of two key components of the model: 1) the compressed drug–cell interaction vector $f_{ij}\in R^{\text{1}\text{2}\text{8}}$, 2) the initial IC50 prediction ${\hat{y}}_{ij}\in R$ by the MLP network. These two are concatenated to form the final vector $Z_{ij}\in R^{\text{1}\text{2}\text{9}}$:


$$Z_{ij}\;=f_{ij}⊕\;{\hat{y}}_{ij}$$ (14)

The final model benefits from the combination of two types of knowledge: deep feature interaction extracted by the neural network, and statistical knowledge extracted by the boosting ensemble (tree-based residual modeling). The XGBoost model is applied to this training vector, using an ensemble of decision trees that incrementally reduce the model error based on the gradient. This model attempts to correct the residual between the actual and the current predicted value. At each iteration *t,* a weighting function $f_{t}\in F$ a decision tree is added to the model so that the prediction function is updated as follows:


$${\hat{y}}_{ij}^{\left(t\right)}={\hat{y}}_{ij}^{\left(t-\text{1}\right)}+f_{t}(zij)$$ (15)

The training process is carried out by minimizing the total loss function, which includes the prediction error and a regularization term to prevent overfitting:


$$\begin{array}{c} L^{\left(t\right)}=\sum {\mathrm{\backslash nolimits}}_{i,j}l(y_{ij},{\hat{y}}_{ij}^{\left(t-\text{1}\right)}+f_{t}(zij))+\Omega (f_{t}) \end{array}$$ (16)

In this equation, *l* is the regression loss function, which in the MoGraphDRP is computed using MSE, and *Ω* is the model complexity term. The additive structure of XGBoost is designed to apply the most accurate correction to the residual error at each iteration by utilizing both the first- and second-order gradients. In the present implementation, the number of trees is set to *T = 100*, the maximum depth of each tree is 6, the learning rate is 0.05, and the subsample ratio is 0.8. These settings are chosen to balance prediction accuracy and overfitting prevention. After training, the final model output is obtained by summing the initial predictions and the corrections made by the trees as follows:


$$\begin{array}{c} {\hat{y}}_{ij}=\sum {\mathrm{\backslash nolimits}}_{t=\text{1}}^{T}f_{t}(zij) \end{array}$$ (16)

The value ${\hat{y}}_{ij}$ is considered the final, refined prediction of IC50 for the drug–cell pair ($d_{j},c_{i}$). As shown in Fig 2, the outputs of the Bilinear module, including ${\hat{y}}_{ij}$ and $f_{ij}$, are concatenated and fed into the ensemble model. The ensemble module, as the final stage of the model, serves as a refinement step to enhance the accuracy and stability of IC50 prediction. This two-stage design combining the representational power of deep learning in the Bilinear stage with the corrective accuracy of tree-based models in the ensemble stage results in improved accuracy, reduced prediction variance, and enhanced robustness of the model under diverse and noisy data conditions.

### 2.5. Implementation and learning procedure

The MoGraphDRP model was implemented using the open-source libraries PyTorch 2.0.1 and DGL 1.1.2 in the Python programming language. All experiments were conducted on a system equipped with an NVIDIA RTX 3090 GPU (24GB) and the Ubuntu 20.04 operating system. To ensure reproducibility of the results, the initial seed was set to 42, and all operations were executed on the GPU indexed as cuda:0.

The dataset was split into 80% for training, 10% for validation, and 10% for testing. This split was performed randomly while maintaining the distribution of IC50 values across each subset. The main network configurations, layers, and optimization parameters are detailed in Table 1.

**Table 1:** Implementation details of the MoGraphDRP method.

Component	Value/ Type
Implementation Framework	PyTorch 2.0.1 + DGL
Type of GNN	GCN
Number of GCN Layers	3 layers
Drug Embedding Size	128
Cell Embedding Size	512
Number of Attention Heads in BAN	4
Dropout	0.4
Activation Function	ReLU
Optimizer	Adam
Initial Learning Rate	1e-4
Batch Size	128
Number of Epochs	200
Learning Rate Scheduler	ReduceLROnPlateau
LR Scheduler Monitoring Metric	Validation loss
Loss Function	MSE
Ensemble Model	XGBoost
Number of Trees in XGBoost	100
Max Tree Depth	6
XGBoost Learning Rate	0.05

To ensure learning stability and normalization of the internal feature distributions, batch normalization was applied in most encoder layers. All key model hyperparameters including the dropout rate, embedding dimension, batch size, learning rate, the number of trees in the ensemble model, and the strategy for integrating multi-omics and drug features were selected empirically through multiple rounds of experiments. For each parameter, several different values and settings were tested, and only those configurations that consistently produced the best validation performance and overall model stability were ultimately used and reported here. In the main model training phase, the Mean Squared Error (MSE) loss function was used for regression. This function is defined as follows:


$$\begin{array}{c} L_{MSE}=\frac{\text{1}}{N}\sum {\mathrm{\backslash nolimits}}_{i=\text{1}}^{N}{\left({\hat{y}}_{i}-y_{i}\right)}^{\text{2}} \end{array}$$ (17)

Where ${\hat{y}}_{i}$ is the predicted value and $y_{i}\;$ is the true drug response value (IC50) for the cell–drug pair, and *N* is the number of samples in each batch. To compute the final loss, the errors of all samples are summed and then averaged, which is considered the final value of the loss function. This method, known as *mean reduction*, ensures that each sample contributes equally to the weight update process, while maintaining the model’s sensitivity to large errors an important aspect in noisy biological data. In the ensemble model, the default *Squared Error Loss* function was used along with $L_{\text{2}}$ regularization to control model complexity and prevent overfitting.

## 3. Results

In this section, the performance of the proposed model is examined from multiple perspectives. First, the utilized datasets are introduced to establish the evaluation framework. Next, the performance metrics are described, followed by a comparison of the obtained results with existing methods. The model’s ability to predict responses to unknown drugs is then analyzed. Finally, a feature importance analysis is conducted to provide deeper insight into the decision-making process of the ensemble model.

### 3.1. Materials and dataset construction

**Fig 4:**
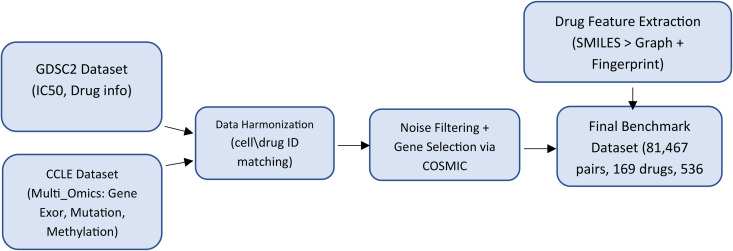
Schematic of the data preparation process.

In this study, version 2 of the GDSC (Genomics of Drug Sensitivity in Cancer) database was used as the primary source for collecting drug response data [36]. This database contains sensitivity measurements such as IC50 values for hundreds of drug–cell line combinations. Initially, the raw dataset included 286 drugs and 969 cancer cell lines. For each pair, the IC50 value was collected on a logarithmic scale. To obtain the molecular features of the cells, the CCLE (Cancer Cell Line Encyclopedia) database [6] was utilized, which provides multi-omics data including gene expression, genetic mutations, and DNA methylation for over 1,000 cancer cell lines. We extracted and integrated the gene expression, mutation, and methylation data for cell lines overlapping with those in the GDSC dataset. To eliminate irrelevant features and reduce noise, only genes listed in the COSMIC (Catalogue of Somatic Mutations in Cancer) database [37] were selected. Genes appearing in the omics data but absent from the COSMIC list were removed. For the mutation data, all mutation types related to a given gene were aggregated to reduce redundancy. As a result of this feature selection process, the input vector dimensions for each omics type were specifically determined: 714 genes were selected for gene expression data, 715 genes for the mutation vector, and DNA methylation was represented using 603 genes. To extract structural features of drugs, we referred to the PubChem database [8]. SMILES strings for each drug were retrieved and converted into molecular graphs. Additionally, drug fingerprints were obtained from Morgan, ESPF, and PubChem sources. During the data cleaning phase, drugs with invalid PubChem IDs or incomplete data were removed. For drugs with multiple identifiers in the GDSC, the version providing the highest IC50 coverage was selected. After the above processing, 536 cell lines and 169 drugs remained, forming 81,467 drug–cell line combinations. This dataset was considered the benchmark dataset. Out of 90,584 possible combinations (536 × 169), approximately 9.6% of the IC50 values were unavailable, and during dataset construction, only those combinations with missing IC50 values were excluded. In the next step, the processed data were used to train the proposed deep learning model. Fig 4 presents a step-by-step diagram of the data preparation process.

The drug response data were obtained from the GDSC2 database and the cellular biological data were extracted from CCLE and aligned with each other. To evaluate the model’s performance on unseen data, the available IC50 values from the CCLE database were also utilized. By excluding combinations already present in the benchmark set, an independent test set was created, consisting of 184 drugs and 8,909 IC50 values for 551 cancer cell lines.

### 3.2. Performance evaluation metrics

To evaluate the accuracy of the model in predicting the drug response of cells to various drugs, a set of standard regression metrics was employed. Each of these metrics captures a specific aspect of model performance and collectively provides a comprehensive view of its accuracy and generalizability. Mean Squared Error (MSE) was used as the model’s primary loss function and measures the average squared deviation of the predictions from the actual values. Root Mean Squared Error (RMSE) was applied to assess the absolute prediction error in the original output unit, and Mean Absolute Error (MAE) evaluates the average absolute distance between predicted and true values. Additionally, the Coefficient of Determination (R²) indicates the proportion of variance in the actual values explained by the model. Pearson Correlation Coefficient (PCC) assesses the linear correlation between predicted and actual values, while Spearman Correlation Coefficient (SCC), a non-parametric metric, measures rank-based correlation.


$$\begin{array}{c} MSE=\frac{\text{1}}{N}\sum {\mathrm{\backslash nolimits}}_{i=\text{1}}^{N}{\left({\hat{y}}_{i}-y_{i}\right)}^{\text{2}} \end{array}$$ (18)

$$\begin{array}{c} RMSE=\sqrt{\frac{\text{1}}{N}\sum {\mathrm{\backslash nolimits}}_{i=\text{1}}^{N}{\left({\hat{y}}_{i}-y_{i}\right)}^{\text{2}}} \end{array}$$ (19)

$$\begin{array}{c} MAE=\frac{\text{1}}{N}\sum {\mathrm{\backslash nolimits}}_{i=\text{1}}^{N}{\left|{\hat{y}}_{i}-y_{i}\right|}^{\text{2}} \end{array}$$ (20)

$$R^{\text{2}}=\text{1}-\frac{\sum_{i=\text{1}}^{N}{\left(y_{i}-{\hat{y}}_{i}\right)}^{\text{2}}}{\sum_{i=\text{1}}^{N}{\left(y_{i}-\overset{―}{y}\right)}^{\text{2}}}$$ (21)

$$PCC=\frac{\sum_{i=\text{1}}^{N}\left({\hat{y}}_{i}-{\overset{―}{\hat{y}}}_{i})(y_{i}-\overset{―}{y}\right)}{\sqrt{\sum_{i=\text{1}}^{N}{\left({\hat{y}}_{i}-{\overset{―}{\hat{y}}}_{i}\right)}^{\text{2}}.}\sqrt{\sum_{i=\text{1}}^{N}{\left(y_{i}-\overset{―}{y}\right)}^{\text{2}}}}$$ (22)

$$SCC=\text{1}-\frac{\text{6}\sum_{i=\text{1}}^{n}\delta_{i}^{\text{2}}}{n(n^{\text{2}}-\text{1})}$$ (23)

### 3.3. Performance comparison of MoGraphDRP Method and existing methods

To assess the accuracy of the MoGraphDRP model, its performance was compared with six advanced models: tCNNs [28], DeepCDR [22], Precily [29], DeepTTA [23], GADRP [30], and BANDRP [26]. These models were previously developed for predicting drug response in cell lines and employed various methods for feature extraction and data integration. All models were trained on a unified benchmark dataset, using an identical data split of 80% for training, 10% for validation, and 10% for testing. The comparison results are reported in Table 2. The evaluation of these results indicates that the MoGraphDRP model outperformed all other approaches across all five primary performance metrics. In particular, the RMSE was reduced to 0.6622, representing a substantial improvement over BANDRP, which achieved an RMSE of 0.9305. Additionally, the MAE was also reduced to 0.4884 in the MoGraphDRP model. In terms of correlation metrics, the MoGraphDRP method achieved the highest concordance with actual values, reaching PCC = 0.9689 and SCC = 0.9524. These results demonstrate that the model’s predictions are not only statistically more accurate but also better aligned with the trends observed in the true data.

**Table 2:** Performance comparison of the MoGraphDRP method with other state-of-the-art approaches on the benchmark dataset.

Method	RMSE	MAE	*R* ^2^	PCC	SCC
tCNNs	1.1574	0.8509	0.8166	0.9042	0.8776
DeepCDR	1.0189	0.7538	0.8579	0.9270	0.9003
Precily	1.1136	0.8345	0.8302	0.9125	0.8792
DeepTTA	0.9634	0.7182	0.8272	0.9105	0.8770
GADRP	0.9953	0.7263	0.8402	0.9298	0.9097
BANDRP	0.9305	0.6769	0.8802	0.9382	0.9168
**MoGraphDRP**	**0.6622**	**0.4884**	**0.9388**	**0.9689**	**0.9524**

The superiority of the MoGraphDRP model can be attributed to its multi-layered and comprehensive design. Firstly, the simultaneous utilization of four cellular multi-omics data sources gene expression, genetic mutations, DNA methylation, and biological pathway enrichment scores provides the model with a broad and multifaceted view of each cell line’s biological state. On the drug side, the integration of two complementary approaches molecular fingerprints and molecular graph structure ensures that drug features are described from multiple perspectives. This combination of low- and high-level representations prevents feature omission and results in a more complete representation of drugs. Moreover, the design of the Bilinear Attention module plays a critical role in accurately modeling drug–cell interactions. Unlike conventional methods that rely on simple feature fusion, this module enables the model to learn bidirectional, feature-level, and nonlinear dependencies between the two independent feature spaces.

Ultimately, one of the main contributors to the improved performance of the model is the incorporation of a refinement stage based on XGBoost at the end of the architecture. In preliminary experiments, the model lacked sufficient accuracy without this component. However, with the addition of this layer, predictions were made more robust against residual errors, and the model’s performance stability was significantly enhanced. This hybrid design leveraging the feature extraction power of deep networks alongside the precision of boosting algorithms has proven particularly effective under complex and noisy biological data conditions.

**Fig 5:**
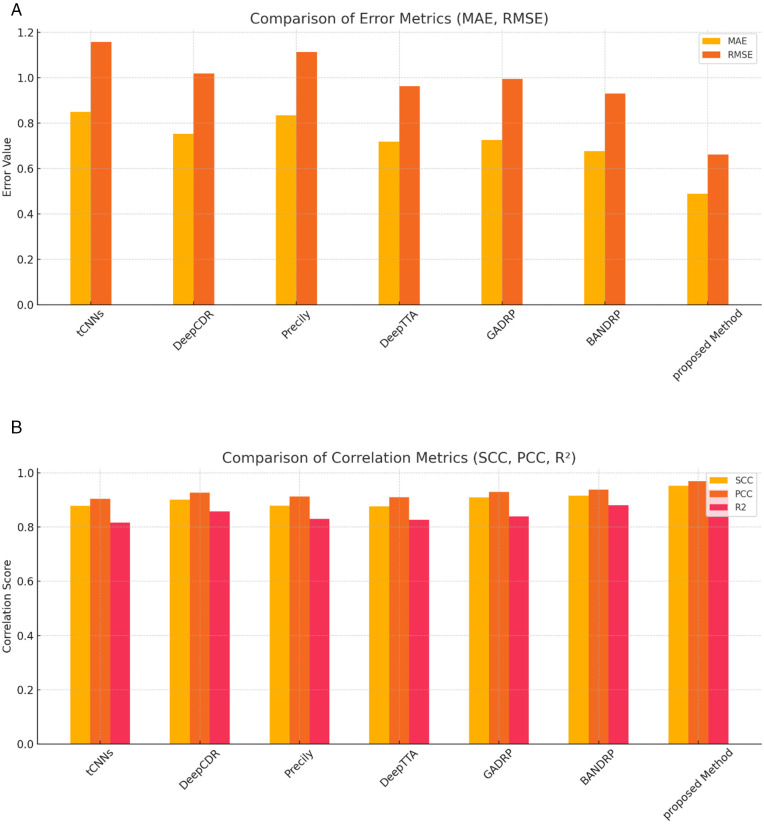
a. Comparison of the performance of different models based on MAE and RMSE metrics on the benchmark dataset. The MoGraphDRP model achieved lower error values in both metrics compared to other methods, indicating higher accuracy in predicting IC50 values. **b.** Performance comparison of different models based on SCC, PCC, and R² metrics shows that the MoGraphDRP model not only achieved the highest linear correlation coefficient (PCC = 0.9689) but also recorded the highest explained variance (R² = 0.9388) among all methods.

To better illustrate the performance differences, Fig 5a and 5b are presented. As shown in Fig 5a, the MoGraphDRP model outperformed the compared methods in terms of RMSE and MAE. This superiority is especially evident in the RMSE metric, which was reduced to 0.6622. In Fig 5b, the values of PCC, SCC, and R² for various models are compared. The MoGraphDRP model clearly surpassed all others across all three metrics, indicating that its outputs not only closely match the true values but also align well with the reference trends and distributions.

**Fig 6:**
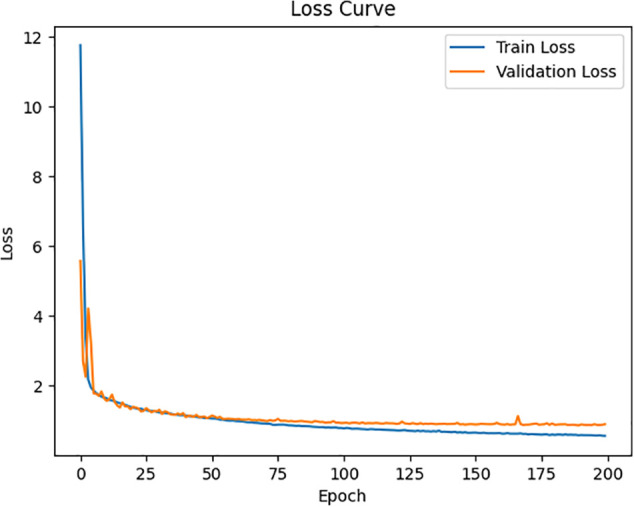
Loss curve over 200 epochs.

**Fig 7:**
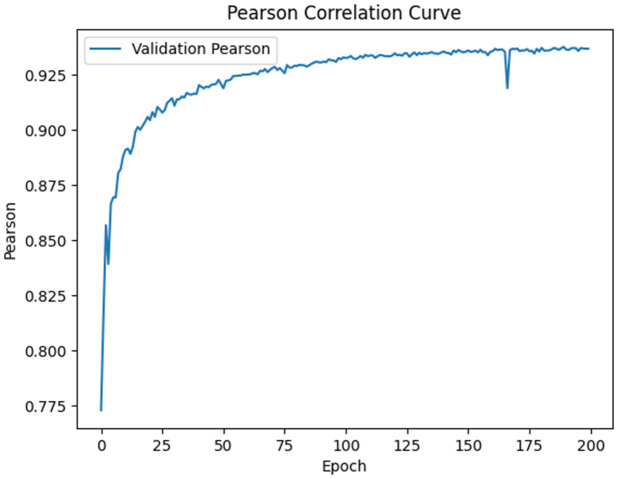
Pearson Correlation Coefficient (PCC) trend on the validation set during training.

To further examine the learning dynamics of the MoGraphDRP model, the values of the loss function and the Pearson Correlation Coefficient (PCC) were recorded and analyzed throughout the training process. As shown in Fig 6, the loss values for both the training and validation sets steadily decreased over time, and from approximately epoch 50 onward, a stable and nearly uniform decline was observed. This trend indicates proper convergence of the model without signs of overfitting. Moreover, Fig 7 illustrates the progression of the PCC score on the validation set. The gradual increase in this coefficient, reaching above 0.96 by the end of training, reflects the model’s improved alignment with the actual values and its effective learning of complex drug–cell interaction patterns. This improvement corresponds to a 28.8% reduction in RMSE and more than 27% reduction in MAE compared to BANDRP, which served as the strongest baseline model.

### 3.4. Prediction of unknown drug responses

**Fig 8:**
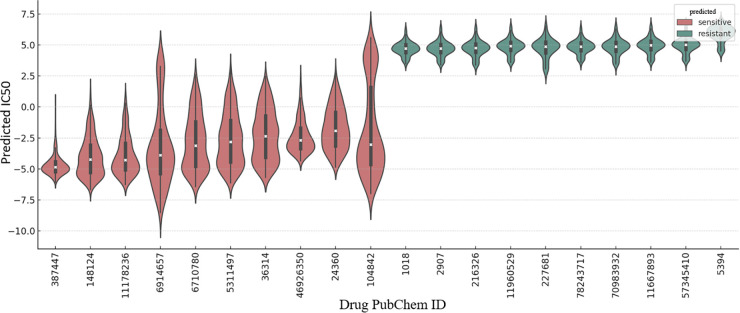
The distribution of predicted IC50 values for the top 10 sensitive and top 10 resistant drugs is shown. This pattern demonstrates the model’s capability to distinguish between active and inactive compounds, even in the absence of direct experimental data.

At this stage, the developed model after being trained on the available IC50 values from the benchmark dataset was utilized to estimate unknown drug responses. Out of 90,584 potential drug–cell line combinations, 81,467 combinations with experimentally measured IC50 values were used as training data. Following training, the model was able to impute 9,117 missing IC50 values. These predictions were organized into a cell–drug response matrix, enabling the ranking of drugs based on their predicted anticancer activity. For this purpose, the mean predicted IC50 value for each drug was computed, and the drugs were ranked accordingly. Fludarabine was identified as the most potent drug candidate. This compound, a nucleoside analog of adenosine, is intracellularly converted into its active triphosphate form and inhibits key enzymes involved in DNA synthesis, such as DNA polymerase and ribonucleotide reductase. As a result, it induces cell cycle arrest and triggers apoptosis in tumor cells [38]. Fludarabine is primarily used in the treatment of hematological malignancies, such as chronic lymphocytic leukemia (CLL). The consistently low predicted IC50 values for this drug across various cell lines support its strong potential as an effective anticancer agent [39]. In contrast, cimetidine was identified as the most resistant drug. Cimetidine is a histamine H2 receptor antagonist commonly used for treating acid-related gastrointestinal conditions [40]. Since this drug does not directly participate in pathways associated with cell division or tumor cell death, its high IC50 value in the predictions appears reasonable [41]. To assess the model’s ability to distinguish between active and inactive drugs in the absence of experimental data, the distribution of predicted IC50 values for the top 10 sensitive and top 10 resistant drugs is illustrated in Fig 8. As observed, the sensitive drugs exhibit a lower and more compact IC50 distribution, while the resistant drugs have a higher and more separable distribution. This supports the model’s capability to accurately predict responses even without empirical data.

**Fig 9:**
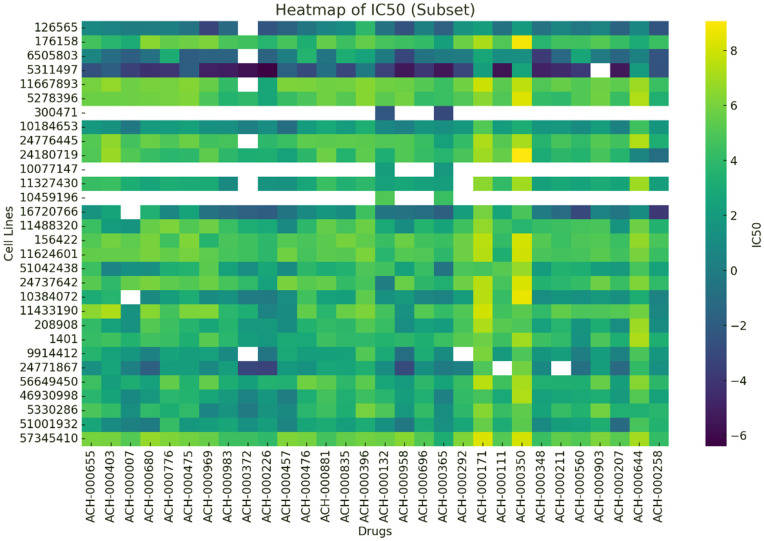
Heatmap of a subset of the predicted IC50 matrix for 30 drugs and 30 cell lines. The color variation indicates the level of cell sensitivity to different drugs. Darker colors represent stronger responses (lower IC50 values).

The conducted analyses demonstrate that the model is not only effective in imputing missing values but also capable of identifying genuinely anticancer compounds among unknown drugs even when such compounds were not present during training. Fig 9 provides an overview of the drug response (IC50) matrix, visually illustrating both the structure of the data and the model’s ability to complete missing entries.

### 3.5. Feature importance analysis

**Fig 10:**
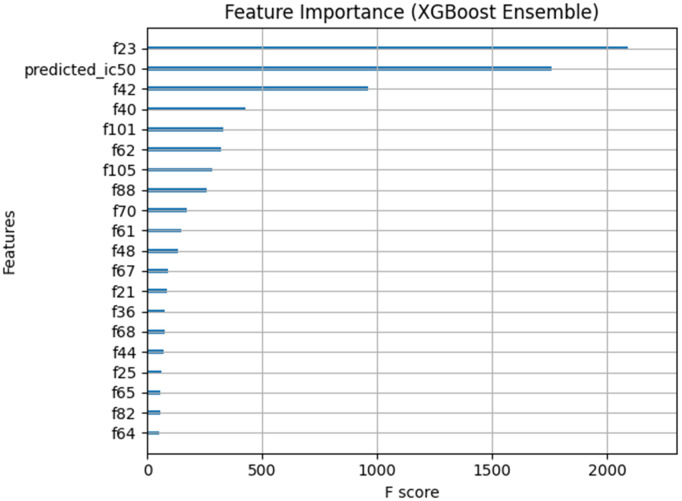
Feature importance scores (F score) of the XGBoost ensemble model. Features *fN* represent dimensions of the Bilinear Attention output vector, and predicted IC50 is the preliminary deep model prediction.

To better understand the behavior of the ensemble model and identify which features contributed most to the final IC50 predictions, the feature importance scores of the XGBoost model were analyzed. The importance of each feature was measured using the F score, which indicates the number of times a feature was used to split nodes in the decision trees. In other words, a higher F score reflects a greater influence of that feature on the model’s decision-making process. As shown in Fig 10, certain dimensions of the Bilinear Attention output vector (the f features), as well as the initial predicted IC50 value (predicted_ic50), had the highest importance in the ensemble model. This demonstrates that both the nonlinear drug–cell interactions modeled by the Bilinear Attention module and the direct output of the deep model play a significant role in improving the prediction accuracy. The feature importance scores for the top 20 features in the XGBoost ensemble model are presented in the figure.

## 4. Ablation study

To analyze the role of key components in the MoGraphDRP model architecture, a series of ablation experiments were designed and conducted. In these experiments, each major module of the model was removed or replaced with a simpler structure to evaluate its exact impact on prediction accuracy. It should be naoted that in all ablation versions, the ensemble module was omitted to assess the base model’s raw performance; the effect of the ensemble is analyzed separately in the following section. In the first set of experiments, the structure of the graph neural network (GNN) was modified to investigate the impact of different GNN architectures on feature extraction from drug graphs. Four widely-used architectures GAT, GIN, GIN +Transformer, and GCN were evaluated. The results in Table 3 indicate that among these architectures, GCN achieved the best performance. However, its accuracy was still lower than that of the final model version. The superiority of GCN can be attributed to its ability to extract stable and effective local relationships, while more complex architectures like GAT suffered from performance degradation due to overfitting in noisy biological data. In the next experiment, the model’s performance was evaluated using each chemical fingerprint Morgan, PubChem, and ESPF individually. The results in Table 4 show that removing any of these fingerprints leads to a significant drop in prediction accuracy. This indicates that each fingerprint provides unique, complementary information about the molecular structure of the drug, and their combined use enables a more comprehensive, multilayered representation.

**Table 3:** Performance comparison of the MoGraphDRP method with different GNN architectures.

Method	RMSE	MAE	*R* ^2^	PCC	SCC
Graph GAT	1.03113	0.7589	0.8571	0.9258	0.8969
Graph GIN	1.1133	0.8458	0.8334	0.9176	0.8953
GIN+TRANSFORMER	0.9670	0.7635	0.8610	0.9310	0.9090
Graph GCN	0.9497	0.6862	0.8788	0.9375	0.9135
**MoGraphDRP**	**0.6622**	**0.4884**	**0.9388**	**0.9689**	**0.9524**

**Table 4:** Performance comparison of the MoGraphDRP method with variations in the drug fingerprint data.

Method	RMSE	MAE	*R* ^2^	PCC	SCC
Only PubChem	1.2551	0.9837	0.7780	0.9063	0.8909
Only ESPF	1.2309	0.9725	0.7865	0.9080	0.8948
Only Morgan	1.1793	0.9175	0.8040	0.9127	0.8955
**MoGraphDRP**	**0.6622**	**0.4884**	**0.9388**	**0.9689**	**0.9524**

To assess the role of cellular biological profiles, each of the four omics data types (gene expression, mutation, methylation, and pathway enrichment) was separately excluded from the model. As shown in Table 5, removing pathway or expression data led to the greatest decrease in R² and PCC metrics. These two data sources provide functional and high-level insights into the cellular state, playing a crucial role in accurately predicting drug response. Mutation and methylation data also contributed meaningfully, but were not sufficient alone. In the final stage, modified versions of the overall model architecture were evaluated. These versions included: complete removal of the graph module, removal of the fingerprint autoencoder, using differently weighted fingerprints, and removing the ensemble. The results in Table 6 indicate that removing the graph module caused a substantial drop across all metrics, underscoring the importance of understanding the drug’s topological structure. Replacing the autoencoder with a simple MLP also clearly reduced accuracy, highlighting the value of non-linear compression in feature quality. The version without the ensemble performed reasonably well but remained inferior to the final model with XGBoost.

**Table 5:** Performance comparison of the MoGraphDRP method with variations in cell line data.

Method	RMSE	MAE	*R* ^2^	PCC	SCC
W/O EXP	1.2203	0.8996	0.7162	0.9127	0.8915
W/O MUT	1.2519	0.9763	0.7335	0.9170	0.8940
W/O METH	1.2968	0.9873	0.7890	0.9235	0.8969
W/O PATH	1.1846	0.9080	0.8010	0.9355	0.8990
**MoGraphDRP**	**0.6622**	**0.4884**	**0.9388**	**0.9689**	**0.9524**

**Table 6:** Performance comparison of the MoGraphDRP method with architectural variations.

Method	RMSE	MAE	*R* ^2^	PCC	SCC
Without graph	2.5642	1.9928	0.1094	0.3314	0.3437
With weighted fingerprints	0.9827	0.7317	0.8699	0.9335	0.9113
Without autoencoder	0.9829	0.7329	0.8642	0.9306	0.9095
MoGraphDRP without XGBoost	0.9497	0.6862	0.8788	0.9375	0.9135
**MoGraphDRP**	**0.6622**	**0.4884**	**0.9388**	**0.9689**	**0.9524**

To select the optimal model configurations, a series of preliminary experiments were conducted with different values of learning rate and batch size. These experiments aimed to identify combinations that, while maintaining training stability, led to the minimum error on the validation set. Ultimately, a learning rate of 1e-4 and a batch size of 128 were chosen as the best settings and were used in all implementations. These experiments demonstrated that the success of the model is significantly influenced by architectural optimization and the precise selection of components, indicating that the MoGraphDRP design is not the result of arbitrary trial-and-error, but rather based on a systematic structural analysis.

### 4.1. Independent test evaluation

To evaluate the generalization capability of the MoGraphDRP model on unseen data, the final experiment was conducted on an independent test set from the CCLE database. To ensure complete independence of this set from the training data, all drug–cell line pairs that were also present in the benchmark dataset (GDSC) were removed. The final set consisted of 8,909 IC50 values for 184 drugs and 551 cancer cell lines, none of which were used during the training, validation, or internal testing phases of the model. The results of this experiment are presented in Table 7.

**Table 7:** Comparison of MoGraphDRP performance on the independent test set before and after ensemble.

Method	RMSE	MAE	*R* ^2^	PCC	SCC
Without XGBoost	0.8334	0.6524	0.7931	0.8979	0.8660
With XGBoost	0.6744	0.5066	0.8860	0.9414	0.8792

As shown by the results in Table 7, adding the XGBoost-based ensemble module led to a significant improvement across all performance metrics. Specifically, the RMSE decreased by approximately 19.7%, while the correlation indices PCC and R² increased noticeably. This enhancement was achieved despite the CCLE dataset being entirely unseen during the model training process. Therefore, the obtained results indicate the high generalization capability of the MoGraphDRP model in cross-dataset scenarios. Moreover, the ensemble module’s role in increasing robustness and reducing systematic errors is clearly validated by this evaluation.

## 5. Discussion

The MoGraphDRP framework in this study presents a comprehensive, biology-driven model for drug response prediction that integrates multi-modal data from both cellular and drug domains, achieving high predictive accuracy. Unlike models that rely solely on a single data type, such as gene expression or linear SMILES representation, this model incorporates four omics profiles (gene expression, mutation, methylation, and pathway enrichment) alongside both graph-based and vectorized representations of drugs. A key innovation of the model is the use of a multi-head bilinear attention module, which enables learning of feature-level, non-linear interactions between drugs and cells. In contrast, previous models predominantly employed simple concatenation or dot-product operations, which are limited in capturing complex biological relationships. Additionally, the inclusion of a refinement stage using the XGBoost algorithm effectively corrects residual errors and enhances the model’s robustness against data noise. Comparison with previous models such as BANDRP, GADRP, and DeepCDR demonstrated that the MoGraphDRP model achieved superior performance across all evaluation metrics, particularly in RMSE and Pearson correlation coefficient (PCC). The reduction in RMSE to 0.6622, compared to 0.9305 in the best prior model, represents an improvement of approximately 28.8%. Ablation studies further confirmed that removing any component of the architecture especially biological pathways or the molecular graph structure led to a significant decline in predictive accuracy, highlighting the importance of simultaneously modeling multiple data modalities.

However, implementation of the MoGraphDRP framework requires access to comprehensive and high-quality omics data, which may not be readily available in some clinical settings. Additionally, the use of graph-based architectures and multi-head attention modules increases computational cost compared to lighter models, potentially necessitating further optimization for large-scale or real-time applications. Moreover, the current architecture focuses on predicting responses to single drugs, and could be extended in future work to support analysis of combination therapies or longitudinal (time-series) data. Another important observation concerns the significant impact of the XGBoost-based ensemble refinement stage. Although the base version of the model alone demonstrated satisfactory performance, evaluations revealed that excluding this final module resulted in decreased predictive accuracy and stability. This hybrid design (deep + ensemble), especially under noisy biological data conditions, enhanced the model’s robustness and generalizability. Similar strategies have also been reported as effective in other studies [3]. Ultimately, the analysis of reconstructed IC50 values for previously unseen data particularly results from the independent CCLE test set confirmed that the model is capable of distinguishing between active and inactive compounds even in the absence of prior exposure. This finding not only underscores the generalization capability of the model but also highlights its potential applicability in clinical settings, such as prioritizing drug candidates for experimental screening. This capability opens the door for using the model in clinical decision-support systems to guide optimal drug selection based on individual patient profiles. Looking ahead, the model can be further enhanced by incorporating Transformer-based encoders, leveraging self-supervised learning strategies on large-scale molecular data, and adapting it to clinical patient datasets such as xenograft models or combinatorial therapies. These advancements could pave the way toward more effective drug design and personalized medicine.

## 6. Conclusion

In this study, a deep, multi-modal, and biologically-informed framework was designed and implemented for the accurate prediction of cancer cell drug responses, which outperformed previous models across all major performance metrics. On the cellular side, four types of biological profiles including gene expression, mutation, DNA methylation, and pathway enrichment were utilized. On the drug side, the integration of molecular graph structure and fingerprint representations, along with the Bilinear Attention module, enabled the model to capture complex feature-level interactions between drugs and cells. Results from extensive evaluations and ablation studies demonstrated that each component of the MoGraphDRP architecture independently contributed to improving predictive accuracy. Moreover, by incorporating an XGBoost-based ensemble refinement module in the final stage, the MoGraphDRP model achieved more accurate and stable predictions especially under noisy biological conditions, where conventional models typically suffer performance degradation. The model’s ability to impute missing IC50 values and to distinguish between active and inactive compounds in previously unseen data underscores its strong generalization capability in real-world and clinical scenarios. These findings suggest that the MoGraphDRP framework is not only architecturally robust and efficient but also biologically interpretable and practically viable for advancement in precision medicine and targeted drug design. Future extensions of this model to temporal data, real patient samples, and combinatorial therapies may establish it as a practical tool for clinical decision-making and personalized treatment planning.
